# Comparison of Embryological Results of Microinjection in Two Groups of Men with and without Requesting Sperm DNA Fragmentation Index Measurement

**DOI:** 10.1155/2024/6769510

**Published:** 2024-01-04

**Authors:** Naser AmirJannati, Arash Mohazzab, Manizheh Fathalian, Hamed Akhavizadegan

**Affiliations:** ^1^Reproductive Biotechnology Research Center, Avicenna Research Institute, Academic Center for Education, Culture and Research (ACECR), Tehran, Iran; ^2^Department of Epidemiology, School of Public Health, Iran University of Medical Sciences, Tehran, Iran; ^3^Urology Department, Tehran University of Medical Sciences, Tehran, Iran

## Abstract

**Introduction:**

The sperm DNA fragmentation index (DFI) is considered a valuable measure to assess male fertility, but the predictive value of DFI for the outcomes in assisted reproductive technology (ART) is still controversial. Therefore, this study is aimed at investigating the effect of requesting a DFI test or performing ART without DFI on the results observed in the embryology laboratory (number of embryos, fertilization rate, and embryo quality) after intracytoplasmic sperm injection (ICSI).

**Methods:**

This retrospective study was conducted on infertile men who underwent ICSI and were referred to the Avicenna Infertility and Recurrent Abortion Treatment Center in Tehran from 2019 to 2022. The samples were categorized into two groups: a case group with DFI measurement and a control group without DFI measurement. We conducted a comparative analysis of the embryology results between the two groups, focusing on parameters such as fertilization rate, number of embryos, and embryo quality. *t*-tests and Mann–Whitney *U* tests were used to conduct single variable analysis. Potential confounding effects were adjusted to use the multivariate linear and logistic regression.

**Results:**

Data analysis showed no significant statistical difference between the case group and the control group in terms of the number of embryos (95% confidence interval for the regression coefficient (*β*) = −0.257‐0.123), and embryo quality (95% confidence interval for *β* = −0.199‐0.114). There was no significant statistical difference between the two groups due to the fertilization rate (95% confidence interval for *β* = −3.42‐3.42), except for the variables of woman's age and sperm count after ICSI, as determined by adjusted linear regression.

**Conclusions:**

Although DFI measurement is used to assess male infertility, its importance as a predictor for the embryology outcomes after ICSI requires further evaluation and the determination of a cut-off point for predicting results. This study was based on retrospectively collected DFI data, and prospective studies confirming the superiority of ICSI outcomes are necessary.

## 1. Introduction

Infertility refers to the lack of fertility after 12 consecutive months of trying to conceive [1]. The worldwide prevalence of infertility is around 15% [2]. Among infertile couples, about 20% of infertility cases include only male factors [3].

The underlying factors of male infertility include genetic and anatomical abnormalities, varicocele, endocrine disorders, infections, systemic diseases, immune factors, environmental toxins, lifestyle, drugs, radiotherapy, and chemotherapy [4]. Assisted reproductive technology (ART), including artificial intrauterine insemination (IUI), in vitro fertilization (IVF), and intracytoplasmic sperm injection (ICSI), has provided new options for infertile couples. Currently, one of the greatest methods for treating infertility is ART. The condition of the sperm and oocytes is one of the many variables that affect these procedures' success rate. The risk of abnormal sperm entering the egg is reduced during spontaneous fertilization and IVF, but it is eliminated after the ICSI procedure, raising questions [5]. Although damaged genome transferred from the father can be processed to some extent during embryonic development, the oocyte's repair system may not be able to fully repair these damages [6]. Low sperm motility, oligozoospermia, and abnormal sperm morphology are the most important factors in semen analysis among male factors of infertility [7].

Around 15% of men who had normal sperm analysis based on the standards of World Health Organization (2010) were recognized as infertile [8]. However, the inability to assess sperm function and the lack of a cut-off point for differentiating fertility are limitations of semen analysis. Therefore, additional markers, such as genetic markers and sperm DNA fragmentation (SDF), are being investigated to overcome these limitations [1].

By the advancement of technology, new methods were developed for detecting sperm chromatin integrity. Male infertility is predicted using the sperm DNA fragmentation index (DFI), which shows the integrity or damage to the sperm DNA. The DFI is calculated as the percent of the spermatozoa with fragmented DNA in relation to the number of all analyzed sperm cells which are defined by sperm nucleus staining [9]. Because sperm DNA passes genetic material to progeny, it is essential to human reproduction. Damage to sperm DNA may have an adverse effect on the results of conception as sperm do not have a repair mechanism [10]. This value was given as a percentage. Studies showed that SDF is an effective predictor of male infertility, but its clinical value in predicting outcomes of microinjection techniques is controversial [11].

One study found that abnormal sperm DNA damage reduces the fertilization rate following IVF, but it does not affect the fertilization rate following ICSI [12]. A meta-analysis study indicated that sperm DNA damage leads to reduced fertilization rates in IVF and ICSI [5]. However, using DFI as a predictor of ART outcomes is still debated. We studied the effect of measuring DFI on fertilization rate, number, and quality of embryos.

## 2. Materials and Methods

### 2.1. Study Design

This retrospective study was conducted on infertile men who visited the Avicenna Infertility and Recurrent Abortion Treatment Center in Tehran from March 2019 to March 2022. The treatment outcomes were analyzed due to the fertilization rate, number, and quality of embryos. The study was approved by the Ethics Committee of Avicenna Research Institute (IR.ACECR.AVICENNA.REC.1401.007). Patients provided consent for their information to be used for research purposes when entering the infertility treatment. The information was collected anonymously. The samples were separated into two groups: a case group that had DFI tested and a control group that did not have DFI measured. Data from patient files were gathered on sperm concentration, motility, morphology, viability, DFI value, number of embryos, number of oocytes in the metaphase stage, embryo grading, and fertilization rate. The relationships among fertilization rate, number, and quality of embryos were compared between the case and control groups.

### 2.2. Patients

The inclusion criteria were at least one year inability to conceive without using any contraceptive method, men aged 25-45 years, absence of infertility regarding female factors, first-time or after one-time failure of ICSI, ICSI performed at the center, at least 5 oocytes in metaphase II, and women with a BMI below 30 kg/m^2^.

The exclusion criteria included genital tract infection at the time of study entry, anatomical abnormalities in the genital tract, such as varicocele and cryptorchidism, systemic diseases or drug treatments in the three months before study entry, recurrent ICSI failure, and wives over 40 years of age.

After checking the inclusion and exclusion criteria and selecting eligible patients within the desired time period, 870 patients were randomly selected. Their data, which included the patient's and his wife's history and demographics, spermogram results, and DFI readings, was logged and input into Excel software. In both the case and control groups, the number, grade, and rate of fertilization of the embryos were examined.

### 2.3. Laboratory Tests

#### 2.3.1. Semen Routine Analysis

Semen samples were collected after 48-72 hours of sexual abstinence, and semen analysis was performed based on World Health Organization guideline manual to determine semen volume, pH, motility, morphology, and sperm concentration. Computer-assisted semen analysis system was used for sperm motility analysis [13].

#### 2.3.2. Sperm DNA Fragmentation Index (DFI) Detection

This test was performed using an SDFA kit (Ideh Varzan Farda, Iran) based on the manufacturer's instructions. In brief, 50 *μ*L of semen was diluted in Ham's F10 medium, and a semen aliquot was mixed with 50 *μ*L of agarose (6.5%). Then, 20 *μ*L of the mixture was loaded onto a pretreated glass slide and placed on a cold surface at 4°C for 5 min. Then, the slides were treated with a denaturizing solution for 7 min and then treated with a lysing solution for 15 min. Following this step, the slides were washed with distilled water for 5 min. The dehydration process included sequential immersion of the slide in increasing concentrations of ethanol (70%, 90%, and 100%), followed by air-drying and staining. A minimum of 200 sperm were examined using a microscope with a magnification of 100x. Sperm exhibiting a substantial or moderate halo were categorized as possessing undamaged chromatin, while those without a halo or displaying a minor halo were categorized as sperm with fragmented DNA. The results were presented as the sperm DNA fragmentation index (DFI) [14].

### 2.4. ICSI Methods

Controlled ovarian stimulation was performed using a standard short GnRH antagonist protocol [15]. For the short-term protocol, a GnRH agonist (Decapeptyl, 0.1 mg) was administered daily. On the second day, gonadotropin (Gonal-F) was initiated, and on the third day, for both protocols, 150 units or 225 units of Gonal-F were given daily for 5 to 6 days, adjusted according to follicle growth and serum estradiol levels. The size and quantity of follicles were assessed by vaginal ultrasonography, FSH, LH, and E2 assays, as well as blood estradiol levels, during subsequent control exams. Human chorionic gonadotropin (hCG) was administered at a dose of 5000–10,000 units once three prominent follicles measuring an average of 17 mm were seen. Specifically, 35-37 hours after injection, eggs were collected under the guidance of vaginal ultrasound. Embryo transfer was performed on the third day after egg retrieval.

### 2.5. Oocyte Fertilization Assessment, Morphology Classification, and Embryo Transfer

In ICSI fertilization, after separating live sperm from fresh semen, good-quality sperm were selected under a microscope and injected into the egg cytoplasm. The pronuclei of the oocytes were evaluated about 16-18 hours after injection to check the success of fertilization. Eggs were considered fertilized when two distinct pronuclei were visible.

The fertilization rate was calculated by dividing the number of embryos by the number of metaphase II eggs and multiplying the result by 100. The fertilized embryos were cultured until the third day. Good-quality embryos on the third day or blastocysts on the fifth day were selected for transfer. Progesterone-supportive treatment was provided to the patients. The term “good-quality embryos” includes embryos with 4 to 6 grade 1 or 2 cells on day 2, 8 to 10 grade 1 or 2 cells on day 3, or well-expanded blastocysts with inner cell mass on day 5, based on Gardner's criteria [16]. One or two embryos with good morphology were selected for transfer on days 3 or 5, while the remaining good-quality embryos were frozen.

## 3. Statistical Analysis

Data were expressed as numbers and percentages for categorical variables, while continuous data were expressed as mean and standard deviation. The data's normality was examined using the Kolmogorov-Smirnov test. For quantitative data that did not have a normal distribution, the independent *t*-test was used, and for data that did, the Mann–Whitney *U* test was utilized. Since the result of ART outcomes might be confounded by DFI test request (confounding by indication), we first performed a logistic regression to evaluate the effect of spermogram indices on DFI test request. Then, we assessed the association of DFI test request variable on ICSI outcomes after adjusting for any potentially confounding factors using linear regression models. A significance level of 0.05 was considered for the *t*-test and Mann–Whitney *U* test. Any interaction between DFI test request and spermogram indices was included in the model if the *P* value was <0.15 for the variable in the linear regression. SPSS version 19 software was used to analyze the results.

## 4. Results

The studied groups were significantly different in terms of male age, female age, sperm volume, and motility C parameters. The results are reported in Table 1.

The mean and standard deviation of men's age in the control group was 36.11 ± 4.14, and in the case group, it was 36.78 ± 3.93 (*P* value = 0.015). The mean and standard deviation of women's age in the control group was 31.90 ± 4.27, and in the case group, it was 32.58 ± 3.97 (*P* value = 0.015). The mean and standard deviation of sperm volume in the control group was 2.83 ± 1.40, while in the case group, it was 2.66 ± 1.44 (*P* value = 0.025). The mean and standard deviation of motility C in the control group was 25.05 ± 7.05, and in the case group, it was 26.41 ± 6.64 (*P* value = 0.003).

The outcome of ICSI before adjusting for the variables showed a statistically significant difference in the fertilization rate between two study groups, but not in the other two factors. The results are outlined in Table 2. The mean fertilization rate in the control group was 26.96 ± 16.70, while in the case group, it was 29.58 ± 16.61 (*P* value = 0.021).

The standard deviation of fertilization rate in the control group was 16.70, and in the case group, it was 16.61. The link between DFI and dependent variables was assessed using logistic regression, although none of the variables showed statistically significant differences. There was no discernible difference between the two groups according to a multivariate linear regression model analyzing the determinants of high-quality embryo, embryo quantity, and fertilization rate while taking DFI measurement and its interactions into account.

## 5. Discussion

This study found that requesting DFI had no effect on microinjection embryology outcomes. Although there was a statistically significant difference in the fertilization rate between the control and case groups, there was no significant clinical difference between the two groups. This difference may be due to the selection of patients based on their indication to request DFI. The study is aimed at eliminating the confounding effect based on the patient's condition using statistical methods.

The relationship between sperm DNA damage and the outcomes of assisted reproductive methods (ART) was studied in several previous studies [7, 8, 17]. Some researchers believe that sperm integrity affects the fertilization rate after ICSI [18]. For example, Speyer et al. found a correlation between sperm chromatin breakdown and a decline in the fertilization rate [19]. High DFI readings have also been linked to adverse reproductive outcomes [20]. Other research, however, has not shown a statistically significant impact of sperm chromatin disintegration on the clinical result after ICSI [8, 21]. The discrepancies in research populations, DNA measuring techniques, and DFI cut-off points might be the cause of this disagreement in findings.

Sperm chromatin testing is not commonly performed as part of male fertility assessment in terms of the lack of a standard protocol for test results, and the validity of threshold. Our limited understanding of DFI and lack of information hinder our ability to accurately understand the relationship between DFI and ART outcomes. Sperm DNA damage occurs during spermatogenesis which can cause damage to chromosomes and loss of sperm integrity. DFI is used to evaluate the extent of DNA damage [22].

Three mechanisms are known to disrupt sperm DNA: abnormal chromatin density during spermatogenesis, aberrant initiation of apoptosis during spermatogenesis or sperm movement, and excessive oxidative stress [23]. There are various methods to measure sperm DNA damage, each showing different aspects of DNA damage [6]. Differences in measurement methods and inconsistencies in the laboratory processes can lead to different results.

This study found that sperm DFI is not a predictor of the fertilization rate, number, and quality of embryos, which is consistent with the results of Sun et al.'s study [24]. This lack of correlation may be due to the optimization of sperm before ART, which can affect fertilization rate and embryo quality. The likelihood of successful fertilization may be increased, and the effect of DNA damage can be decreased by choosing the best sperm for fertilization and screening for high-quality sperm [25, 26]. The fact that oocytes may repair sperm DNA damage has also been documented in other research [27, 28], which might account for the absence of association between DFI measurement and ICSI results.

The variations in DNA damage measurement methods, protocols, and differences in the study population, as well as the study method, have complicated the drawing of definitive conclusions. Although the degree of DNA damage as a male factor can have an adverse effect on fertility outcomes, the extent to which DNA damage can predict ICSI outcomes is still controversial. Our understanding of its measurement and effects is still unclear, and our knowledge of DNA damage is incomplete [5, 6].

As a retrospective study, one of the limitations of this study is the lack of some clinical and laboratory information. Future studies should consider conducting prospective randomized studies to obtain more accurate results.

## 6. Conclusion

Based on the results of our study, the request for DFI test is not strongly correlated with the embryology results following ICSI in patients without a history of recurrent failure.

Therefore, DFI test alone is not a powerful tool to predict the embryological results of microinjection. While the DFI value is a valuable tool to evaluate the male fertility in clinical sperm testing, further studies are needed to understand its significance as a predictor of fertilization outcome following ICSI. It does not seem required to do regular DFI testing in infertile males. However, because of this test's affordability and ease of use, it may be recommended in situations when couples that meet the required indications succeed only sporadically or completely.

## Figures and Tables

**Table 1:** Comparison of baseline characteristics between two study groups.

	Control (*n* = 435)				Case (*n* = 435)				*P* value^∗^
	Mean	SD	Min	Max	Mean	SD	Min	Max	
Age (male)	36.11	4.14	25.00	45.00	36.78	3.93	25.00	45.00	0.015
Age (female)	31.90	4.27	20.00	39.00	32.58	3.97	20.00	40.00	0.015
Volume	2.83	1.40	0.20	9.00	2.66	1.44	0.20	8.00	0.025^∗∗^
Count (10^6^)	85.17	67.37	0.63	528.00	87.84	76.08	1.00	490.00	0.583
Concentration (10^6^)	30.87	17.96	0.25	129.00	33.39	22.39	1.00	135.00	0.068
Morphology	1.17	1.25	0.00	5.00	1.03	1.20	0.00	9.00	0.103
Motility A	1.27	3.67	0.00	53.00	0.91	2.02	0.00	15.00	0.507^∗∗^
Motility B	25.17	9.05	0.00	40.00	24.29	9.16	0.00	40.00	0.156
Motility C	25.05	7.05	0.00	45.00	26.41	6.64	5.00	45.00	0.003
Motility D	48.45	12.95	20.00	100.00	48.40	12.80	22.00	93.00	0.950
Vitality	88.65	7.16	20.00	99.00	88.87	7.10	40.00	96.00	0.641

^∗^ *P* value was reported using independent sample *t*-test. ^∗∗^*P* value was reported using the Mann–Whitney *U* test.

**Table 2:** Unadjusted comparison of ICSI outcome compared between two study groups.

	Control (*n* = 435)				Case (*n* = 435)				*P* value
	Mean	SD	Min	Max	Mean	SD	Min	Max	
Fertility rate (%)	26.96	16.70	2.27	100	29.58	16.61	5	100	0.021
Embryo number	3.3218	1.50034	1.00	10.00	3.3908	1.31068	1.00	9.00	0.470
High-grade embryo number	2.64	1.18	0	8	2.68	1.14	0	8	0.579

## Data Availability

Data is available upon request.
